# A nutritional blend of taurine, vitamins B6, B9, and B12 improves motivated behaviors in healthy adults—a double-blinded randomized clinical trial

**DOI:** 10.3389/fnut.2026.1711478

**Published:** 2026-03-11

**Authors:** Veeda Michelle Anlacan, Roland Dominic G. Jamora, Laura-Florina Krattinger, Evelina De Longis, Mickaël Hartweg, Myriam Steinmann, Laura Trovò

**Affiliations:** 1 Department of Neurosciences, College of Medicine and Philippine General Hospital, University of the Philippines Manila, Manila, Philippines; 2 Clinical Research Unit, Nestlé Research, Lausanne, Switzerland; 3 Nestlé Institute of Health Sciences, Nestlé Research, Lausanne, Switzerland

**Keywords:** antioxidant, mental performance, motivation, taurine, B vitamins

## Abstract

**Introduction:**

Motivation is a key driver in achieving goals and performing daily tasks, involving cost–benefit valuations of the amount of effort required for a particular reward and can be influenced by socio-environmental factors and neurological conditions that may impact the brain reward circuitry. Notably, research has shown that higher glutathione levels (GSH) in the nucleus accumbens are linked with better and more consistent performance in effortful tasks in both preclinical models and humans.

**Methods:**

Building on these findings, we identified candidate nutrients found in foods that could enhance brain GSH production as a possible approach to sustain motivated behaviors. In primary astrocytes in vitro, we discovered that taurine was able to efficiently increase GSH production and protect mitochondria from oxidative stress damage, but only when levels of vitamin B9 were adequate. The above led us to test a blend of taurine, vitamin B6, B9, and B12 in humans, in a randomized, double-blind, 2-arm, cross-over study with 44 participants aged 25–40 years old. We assessed the impact of four-week supplementation of taurine, vitamins B6, B9, and B12 on effortful motivated behaviors. Motivational performance was measured by the Monetary Incentive Delay Task coupled to a physical effort after 14 days and 28 days of supplementation.

**Results:**

Results showed significant improvements after 14 days supplementation in the first period, as well as after 28 days in the second administration period, compared to placebo. Notably, when receiving the active blend, participants showed a consistent motivational performance throughout the task. The blend was also found to reduce the number of lapses during the Psychomotor Vigilance Task after 14 days, but not after 28 days of intake.

**Discussion:**

Overall, these findings demonstrate how targeted nutritional supplementation can sustain brain health and modulate behaviors, such as motivated and goal-oriented performance.

**Clinical trial registration:**

https://clinicaltrials.gov/study/NCT05733364, NCT05733364.

## Introduction

Motivated behavior is fundamental to our daily lives, driving us to take action and achieve our goals. Motivation involves a complex process of evaluating the costs and benefits associated with actions, wherein individuals weigh the level of effort one is prepared to invest against the potential rewards or benefits that may be gained as a result. This evaluative process is essential, as it influences our decision-making and ultimately determines the actions we choose to pursue (1). Furthermore, motivation is not merely a mechanism to drive behavior; it plays a vital role in achieving personal goals and enhancing overall well-being (2–4).

Notably, there are significant variations in motivated behavior among healthy individuals, as seen in differences in engagement with effortful activities and variations in brain activity during laboratory tasks (5, 6). Motivational deficits, such as apathy, anhedonia, or anergia, are commonly observed in various brain disorders (7–10) in aging and obesity (11–13). Understanding the neurobiological mechanisms underlying these individual differences is essential, as it can inform targeted interventions to enhance effortful performance.

Key areas such as the prefrontal cortex and the striatum, including the nucleus accumbens, have been shown to be critical in decision-making and motivational behaviors (14). The nucleus accumbens is considered as a neural interface between motivation and action, having a key role in food intake, sexual behavior, reward-motivated behavior, stress-related behavior and substance dependence (15). In previous studies, the connection between effort-based motivated performance in humans and levels of various metabolites in the accumbal region, was measured using 1 H-MRS at 7 Tesla before performing a physical effort-based motivated task (MIFT) (16, 17). The glutamine-to-glutamate ratio predicts better overall performance and specifically relates to stamina (16). Furthermore, Zalachoras et al. (17) demonstrated that glutathione (GSH) levels correlate with enhanced motivated performance in young adults within the same behavioral paradigm. Using preclinical models, a causal link between GSH levels in the nucleus accumbens and performance was established, showing that both local and systemic modulation of GSH can influence motivated performance and stamina (17).

Through a screening of 17 nutrient candidates, including vitamins, amino acids and bioactive compounds, and their combinations (data not shown), alongside existing literature (18), we identified a blend of taurine, vitamins B6, B9, and B12 as potential enhancers of brain GSH levels. Taurine, a free amino acid abundant in various tissues and especially concentrated in the central nervous system (19, 20), is essential for optimal brain function. In humans, taurine levels can fluctuate between 1 and 20 μmol/g, with plasma concentrations typically ranging from 42 to 156 μmol/L. While taurine is primarily obtained through dietary sources such as meat, seafood, and eggs, it can also be synthesized endogenously in the liver, brain, heart, and lungs. The synthesis of taurine begins with the amino acid cysteine, which is a crucial precursor for GSH production. Ensuring adequate dietary intake of taurine can enhance cysteine availability, thereby facilitating GSH synthesis.

Research has shown that taurine supplementation can elevate GSH levels, including in the brain. For instance, in a rodent model of age-related disease, animals fed a diet containing 2.5% w/w taurine for two months exhibited increased GSH levels in whole brain homogenates (21). Additionally, both low and high doses of taurine (100 and 400 mg/kg via oral gavage) over nine weeks increased GSH in brain homogenates of mice exposed to D-galactose (22) and improved cognitive deficits in the aging model used in that study. Other preclinical studies suggest that similar doses may induce mild anxiolytic effects (23, 24) which could be beneficial to motivated behavior.

**Figure 1:**
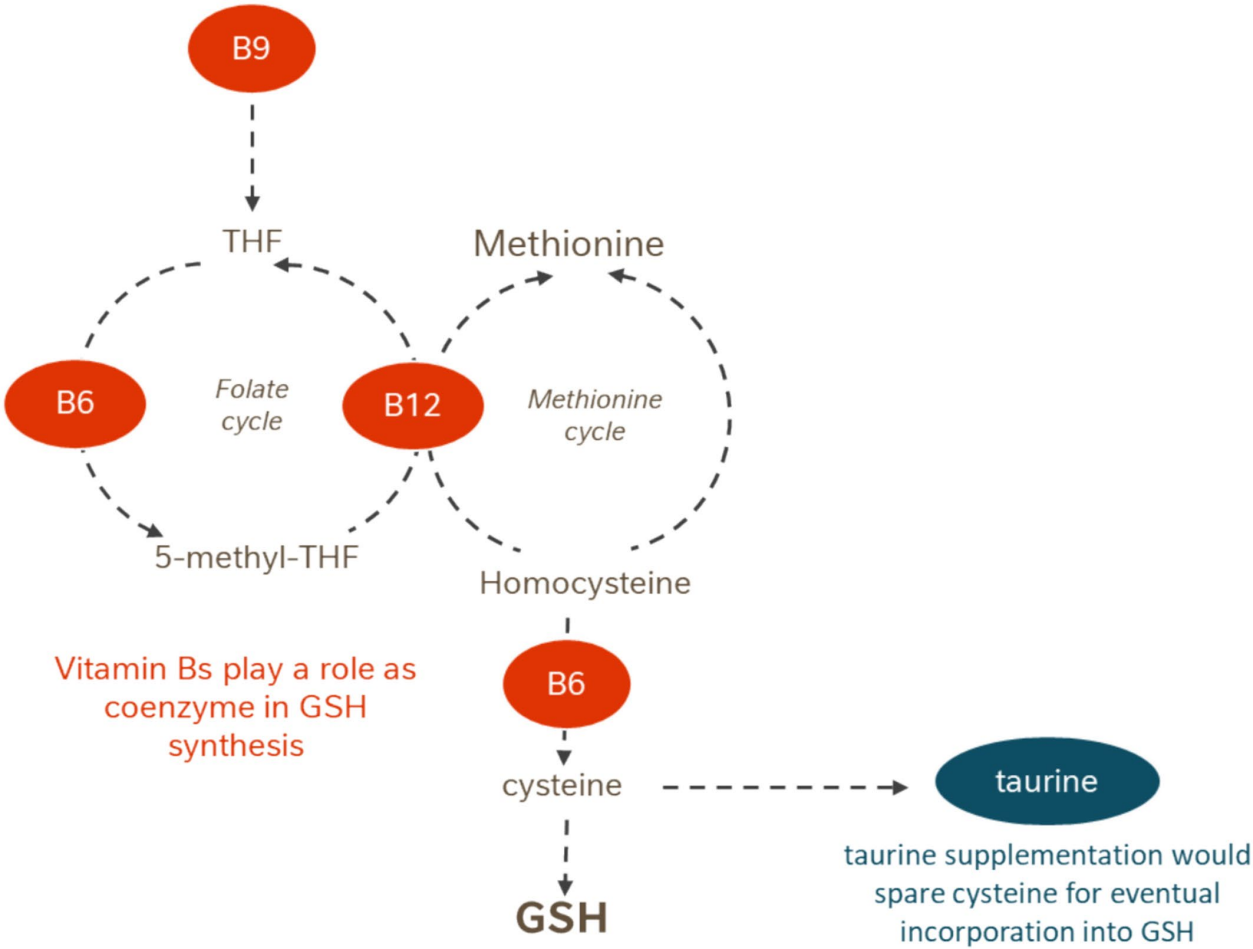
Scheme of the involvement of taurine and vitamin B6, B9, and B12 in the synthesis of glutathione. Glutathione acts also as glutathione peroxidase (GPx) essential substrate in cellular antioxidant defense against lipid and protein oxidation.

Within the same synthetic pathways for GSH production, adequate levels of several B vitamins are essential, as they serve as enzymatic cofactors in multiple metabolic pathways contributing to GSH synthesis: (i) vitamin B6 contributes to normal cysteine synthesis, regarded as the rate-limiting factor in GSH synthesis; (ii) vitamin B9 supports amino acid synthesis, including the conversion of serine to glycine, one of the three amino acids that constitute GSH; and (iii) vitamin B12 is essential for normal homocysteine metabolism, thereby regenerating methionine and facilitating its cycle upstream of GSH synthesis (see Figure 1).

Nutritional deficiencies can significantly impair physiological functions, while adhering to recommended dietary intake levels can help restore and even enhance these functions. Increasing evidence underscores the critical role of diet and nutrition, not only in supporting physical health and body composition but also in promoting brain health. Nutrition indeed plays a significant role in cognitive and mental well-being, and the relationship between nutrients and the regulation of brain metabolites may represent one of the mechanisms through which nutrition influences cognitive function (25, 26). Consequently, gaining insight into how key brain metabolites, such as GSH, influence behaviors and how nutrition can enhance these processes may offer a novel approach to developing effective dietary supplementation strategies to support and optimize cognitive and behavioral outcomes. The aim of this work was to develop and test a nutritional blend to promote motivated behavior, by enhancing GSH levels. To this end, we conducted first an *in vitro* study to develop an optimal blend of taurine and B vitamins able to facilitate GSH production. Subsequently, we tested in a randomized clinical trial the efficacy of this blend in sustaining motivated behavior among healthy adults.

## Methods

The *in-vitro* study methods are available in the Supplementary material.

### Clinical trial objectives

The purpose of the clinical study in humans was to evaluate the efficacy of four weeks supplementation of a combination of vitamin B6, B9, B12, and taurine on motivated behavior, as a primary outcome, and on attentional performance, and self-reported mood (e.g., fatigue, vigor) as secondary outcomes. To evaluate the effect of the intervention on levels of B vitamins and taurine, blood markers were included as exploratory outcomes.

### Design

The trial followed a randomized, double-blind, placebo-controlled, crossover design with two arms (ClinicalTrials.gov, NCT05733364, 19.01.2023). Each administration period lasted 28 days (±1 day), separated by a washout period of 28 days (±1 day).

### Participants

The study involved 45 healthy adults, males and females, and was conducted at the Philippine General Hospital, Manila. To be eligible, participants had to be between 25 and 40 years of age, to be healthy as per site physician medical assessment, to be working for 8–12 h per day, and to have a body mass index (BMI) between 18.5 and 27.5 kg/m^2^. The study excluded individuals who consumed alcohol or caffeine beyond recommended daily limits, those who smoked, were pregnant or lactating, had used supplements or energy drinks with taurine or B vitamins in the 30 days before enrollment, or suffered from sleep disorders. The full list of exclusion criteria is available in the Supplementary material.

### Sample size calculation

The sample size calculation was based on previous research examining the association between levels of glutathione and motivation (17), and the performance of the relevant population in the MIFT (6, 16). In order to detect a medium effect size of 0.625 (average accuracy increase of 0.08 and standard deviation of 0.128) for the primary outcome (MIFT accuracy), with an intra subject correlation of 0.3, a power of 80% a false positive rate set to 5%, and assuming a 30% dropout rate, the target sample size was 44 participants. A protocol planned interim analysis was conducted for this cross-over study when 12 participants completed the first administration period (period 1), in order to re-assess the sample size based on the primary outcome data. The study team was kept blinded to the results of the interim analysis and the data monitoring committee recommended pursuing the study as originally planned, i.e., without further inflation of the sample size. Results from stage 1 (prior to interim) and stage 2 (post-interim) were combined using the inverse normal method and the median unbiased estimate calculated.

### Test products

The test product contained active ingredients (taurine, vitamin B6, B9, and B12) as well as excipient (132.7 mg microcrystalline cellulose). The vitamins were supplied through a vitamin premix to ensure the following dosage was provided per capsule:

- taurine: 500 mg
- vitamin B6: 1.3 mg
- vitamin B9: 0.2 mg
- vitamin B12: 2.4 mcg

The placebo product consisted of capsules visually identical to the test product, but containing 100% microcrystalline cellulose (337 mg).

The products were provided in a bottle of 30 capsules (including 2 leftovers) to cover the consumption period of 28 consecutive days (±1 day).

### Procedures

**Figure 2:**
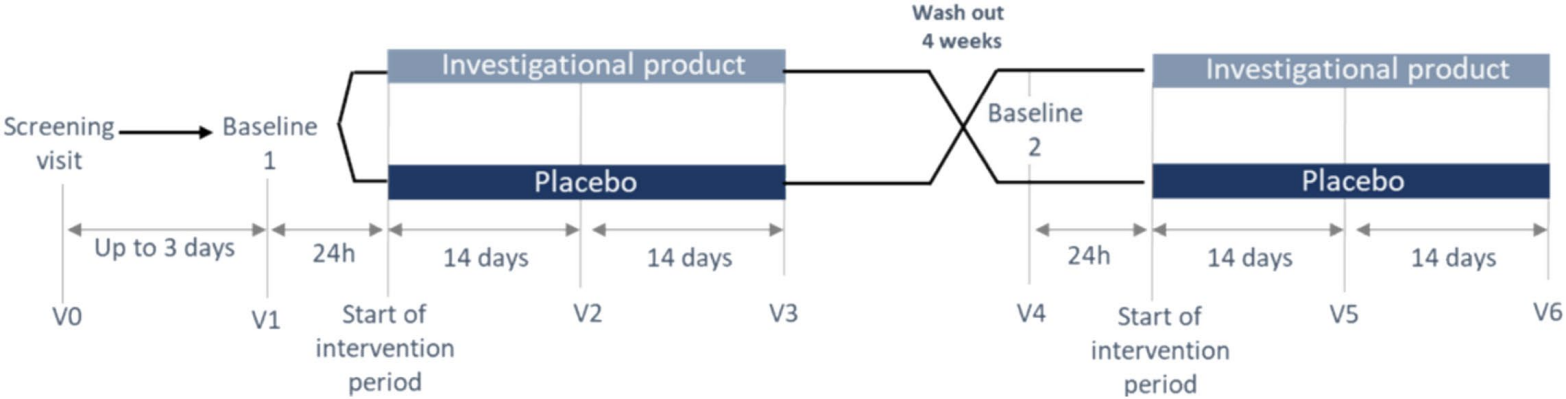
Overview of the study periods and visits.

The trial consisted of a first visit for participants screening and familiarization with the set-up, instructions and equipment, followed by two administration periods lasting 4 weeks. For each administration period, 3 visits were planned (1 baseline and 2 testing visits 14 days ± 1 day apart) for a total of 6 visits (see Figure 2).

During the baseline visit (V1) and during the final visit of each intervention period (V3 and V6), participants underwent a blood draw to determine levels of taurine and B vitamins. To match the testing conditions during all visits, participants were asked to have a light breakfast (e.g., maximum 2 bread rolls, or 1 cup of rice and one egg or 1 small sausage). They were also asked to not consume any caffeinated beverages before coming to the lab, and to avoid strenuous exercise within 24 h of scheduled assessment days. After the blood draw, participants consumed a standardized snack (e.g.; a specific fruit such as one mandarin orange, or one local citrus dalandan) and waited approximately 30 min before filling out mood and motivation questionnaires and performing motivation and cognitive tasks. Primary and secondary outcomes were measured at all timepoints (i.e., V1, V2, V3, V4, V5, and V6). The tasks and the questionnaires are described in the “Measures” section.

The products for the entire intervention period were provided at the end of each baseline visit, to be consumed once during the daytime with a glass of water and the standardized snack to increase absorption of vitamin B12. Compliance was measured by asking participants to report the time and conditions of the investigational product consumption on a paper diary and assessed during the site visits. For each period, participants were considered compliant if they fulfilled ≥23 days of intake; ≤2 consecutive missed/interrupted intake days and confirmed intake on days of assessment (V2 and V3 for period 1; V5 and V6 for period 2).

Each product kit was coded by the manufacturer with unique numbers. The assignment of each participant to test product or comparator was done randomly using integrated eCRF randomization. Correspondence between blinding product codes and investigational product assignment was kept confidential and maintained blinded to personnel and other parties involved in conduct of the trial until the database was locked for final analysis.

The randomization to sequences was stratified by sex and performed dynamically by the minimization technique using the dynamic allocation algorithm, second-best probability set to 15%, with the help of the Medidata-RTSM software.

From enrolment and for the duration of the study, participants were able to report possible adverse events and intake of concomitant medication/supplements. A week after Visit 6, participants were contacted by phone to ensure that they had not experienced any adverse events since the last visit.

The study protocol received ethical approval from the institutional review board (UPMREB CODE: 2022-0425-01). An informed consent form was obtained for all participants enrolled in the study.

### Measures

#### Motivated performance

To objectively measure levels of motivation, we used the modified Monetary Incentive Delay Task (MIDT) as an incentivized effort-based test (henceforth, Monetary Incentive Force Task, MIFT) (6, 16, 17, 27). Participants were asked to exert the established force (50% of the individual baseline maximum capacity MVC force) on a hand dynamometer (TSD121B-MRI, Biopac) within a specific timeframe (e.g., 2 seconds) and sustain it for 3 seconds after. Participants were seated in front of a computer screen at 90 cm distance and were instructed to keep the same right upper limb position (i.e., upper arm and forearm at 90° angle and hand extended) whenever using the dynamometer (see Supplementary Figure 3A). Trials started with a projection of a fixation cross followed by an anticipatory signal (3 seconds) indicating the trial’s incentive (10, 20, 50 pesos, or null). The beginning of the force exertion period was signaled by the appearance of a red circle around the fixation cross. If the established threshold was reached within 2 seconds, the red circle was replaced by a green circle. The green circle also indicated that participants had to maintain the contraction force level above the threshold for 3 more seconds. If participants did not reach the threshold in the initial 2 seconds or if the force level fell below the maintenance threshold during the 3 seconds maintenance period, the trial was considered failed and visualized by a red cross occurring on the screen. If the force was maintained for the required 3 seconds, a green tick indicated successful task performance during a single trial (see Supplementary Figure 3B). Their capacity to perform the action and exert the necessary effort within the specified timeframe was used as an index of motivational performance (6, 17, 27). The mean change from baseline and across sessions in success rate in achieving rewards through effort exertion was used to evaluate changes in objective motivation. As in previous studies, the MIFT task was conducted in two sessions, with a three-minute break between them. The data analyzed by blocks served as an indicator of stamina, which reflects the ability to maintain stable performance from beginning to end.

#### Attentional performance

A 10 min Psychomotor Vigilance Test (PVT) (28) was used to detect changes in the capacity to sustain attention. Participants were asked to respond as quickly and accurately as possible by pressing a keyboard button to visual stimuli appearing in a random frequency (2–10 s presentation interval). Outcome measures included omission rate over the total number of trials presented (lapses) and mean reaction time.

#### Perceived workload

Levels of perceived workload after performing demanding tasks were assessed by using the NASA Task Load Index (NASA-TLX) (29). This questionnaire evaluates six dimensions of perceived workload: perceived mental, physical, and temporal demands, self-perceived overall performance, levels of perceived effort exertion and levels of frustration.

#### Mood (fatigue and vigor)

The efficacy of the intervention on self-reported mood was evaluated by using the Profile of Mood States Short-Form 2 (POMS-SF 2). POMS-SF 2 (30) is a questionnaire consisting of 37 items that load into 6 mood constructs: tension/anxiety, depression/dejection, anger/hostility, fatigue/inertia, confusion/bewilderment, and vigor/activity. The two domains of interest for the present studies are fatigue/inertia and vigor/activity.

#### Blood measurements

To ensure the integrity of the specimens and minimize cross-contamination, a standardized specimen collection protocol was followed. Specimens were collected in a specific order using designated tubes, as outlined below.

Serum Separator Tube (SST II): 3.5 mL of whole blood was collected for the analysis of vitamins B9 and B12. Lithium Heparin Tube: 4 mL of whole blood was collected for the analysis of taurine. EDTA Tube: 2 mL of whole blood was collected for the analysis of vitamin B6. Microcentrifuge Tubes: 1 mL of serum from the SST II was collected for the analysis of vitamins B9 and B12. 0.5–1 mL of plasma from the EDTA tube was collected for the analysis of vitamin B6. 1 mL of plasma from the Lithium Heparin tube containing preservative was collected for the analysis of taurine. Each tube was gently inverted 8–10 times to ensure proper mixing of the blood. Each tube was labeled with the subject’s ID, visit number, date, and time of collection. The SST tube was placed in a vertical position at room temperature for a minimum of 30 min and a maximum of 1 h to allow for clotting. Following the clotting period, all specimens were centrifuged for 10 min at 1,300 RCF. All samples were dispatched to MetaMetrics Laboratory in frozen condition (−20 °C) every two weeks and stored for no more than 1.5 months before analysis. Each specimen/sample was examined for abnormalities, including icteric, lipemic, lysis, and turbidity. It was ensured that pre-analytic, analytical, and post-analytical validations were completed satisfactorily. The results of control samples were verified to ensure they fell within the specified control range. The control specifications for each test are as follows:

- vitamin B6 (plasma): Immundiagnostik/KC2100KO (CE-IVD certified)
- vitamin B9 (serum): Immundiagnostik/KIF005CTRL (CE-IVD certified)
- vitamin B12 (serum): Immundiagnostik/KIF012CTRL (CE-IVD certified)
- taurine (plasma): ERNDIM/AMI-02.1 and AMI-02.2 (ERNDIM IQCS)

If necessary, repeated inspections and control testing were conducted.

### Statistical analyses

The clinical data analysis was performed using R version 4.2.1.

Carry-over and period effects were investigated by comparing the baseline measurements at the beginning of each period (V1 and V4) as well as interpreting the beta coefficient from the interaction between the period and the assigned treatment. In the presence of either a carryover effect or period effect, a repeat of the analyses focusing on data from the first period (prior to washout) was planned. Analyses were conducted on all randomized participants.

Mixed linear models were used to evaluate the changes in blood levels of vitamins B6, B9, B12, taurine, motivated behavior, performance of attention, mood and perceived workload as a function of the product and time (day 14 ± 1 day or to day 28 ± 1 day). In all models, the following fixed covariates were entered: assigned treatment (investigational product or placebo), baseline measurement of the outcome of interest, visit (day 14 ± 1 day or day 28 ± 1 day), visit in interaction with treatment, period (period 1 or period 2), sex and, for MIDT outcomes, incentive level. The participant identification number was included as a random effect to account for within-subject variability. Log transformation was performed as needed to meet the normality assumption. Robust mixed models were used to analyze the PVT data due to the presence of outliers.

Statistical tests are based on two-sided Wald tests using Kenward-Roger degrees of freedom when comparing pairs of estimated marginal means extracted from the linear mixed model. Statistical significance is tested at 5% level, with no correction for multiple testing. We illustrate significance in the figures of this manuscript with the following conventions: *p* ≤ 0.05 is denoted with *, *p* ≤ 0.01 is denoted with ** and *p* ≤ 0.001 is denoted with ***.

## Results

### *In vitro* testing of GSH production modulation

We explored how nutrients can modulate and support GSH production in rat astrocytes. Astrocytes are thought to have a more efficient GSH system than neurons. In-vitro studies suggest that they not only have much higher levels of GSH immediately available, but also much higher activities of the crucial enzymes involved in GSH metabolism (31, 32) and levels of important intermediates (33). Thus, astrocytes represent an excellent model to investigate GSH production due to their higher GSH levels and enzymatic efficiency. As the medium is usually high in B9 (about 9 mM), we simulated a state of nutrient inadequacy by utilizing a depleted medium with reduced levels of vitamin B9 sourced only from the added serum (34). The data revealed that, when B9 levels are insufficient—an occurrence frequently observed in the population—taurine alone does not effectively elevate GSH levels. However, when vitamin B9 is supplemented, taurine demonstrates significant efficacy (*p* = 0.001 taurine vs. taurine + B9 1:2,500; *p* = 0.002 taurine + B9 1:700; see Supplementary Figure 1). Consequently, we have identified an optimal ratio for taurine, which is 2,500 times the amount of B9.

Mitochondrial metabolism is required to continuously adjust to stress or challenging conditions to sustain the bioenergetic levels essential for cellular functions. This metabolic adaptation utilizes the spare capacity of mitochondria to fulfill increasing energy demands; however, oxidative stress, which is a normal process and byproduct of brain functioning, reduces this spare capacity, limiting the ability to meet heightened needs (see Supplementary Figure 2A). As illustrated in Supplementary Figure 2B, also the coupling efficiency deteriorates in the presence of oxidative stress. Tightly coupled mitochondria produce more ATP while utilizing less substrate and generating minimal heat dispersion. In contrast, loosely coupled mitochondria yield less ATP, requiring more substrate and resulting in greater heat dispersion due to proton leakage. In Supplementary Figures 2C,D, a taurine to vitamin B9 ratio of 1:2,500 demonstrated protective effects on mitochondria against oxidative stress by enhancing their spare capacity (taurine vitamin B9 1:2,500 vs. taurine *p* = 0.020: taurine vitamin B9 1:2,500 vs. taurine vitamin B9 1:700 *p* = 0.060) and coupling efficiency (taurine vitamin B9 1:2,500 vs. taurine *p* = 0.034: taurine vitamin B9 1:2,500 vs. taurine vitamin B9 1:700 *p* = 0.017). The 1:2,500 ratio, however, shows superior efficacy, outperforming the alternatives up to the highest dose. Taurine and vitamin B9 ratio 1:2,500 also improved coupling efficiency in a dose-dependent manner. This enhancement supports optimal ATP production while minimizing energy dispersion after oxidative stress exposure.

### Clinical study participants

**Figure 3:**
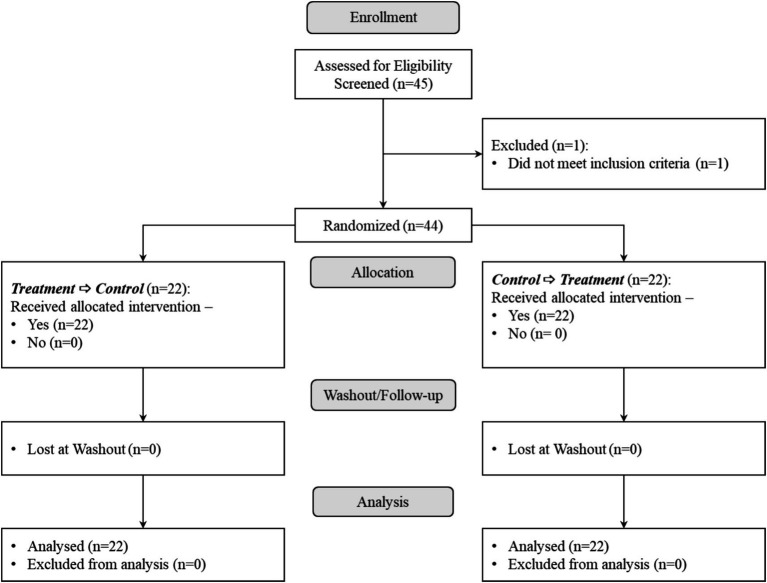
CONSORT study flow diagram.

Forty-five subjects were screened for the trial and 44 (48% females) were enrolled (1 screening failure due to higher BMI, see Figure 3) between March and July 2023. Execution of the trial was completed in October 2023. Participants’ characteristics are reported in Table 1. Four subjects reported authorized concomitant medications (see Supplementary Table 2). The protocol was applied in a qualitative way, with 13 participants reporting at least one protocol deviation and 40 subjects without major protocol deviation. The adverse events reported by 25 participants were all mild and mostly related to the study procedure (i.e., momentary pain on hand grip due to MIFT hand grip dynamometer; see Supplementary Table 3).

**Table 1:** Participants characteristics (*N* = 44).

Variable	Total group		Active first		Placebo first	
	Mean (SD)	Range	Mean (SD)	Range	Mean (SD)	Range
Age	31.7 (4.16)	25–40	31.5 (3.60)	25–39	31.9 (4.74)	25–40
Height (cm)	160.9 (8.59)	139.7–177.8	160.1 (8.42)	139.7–172.7	161.6 (8.89)	144.8–177.8
Weight (kg)	60.9 (10.55)	43.6–83.3	60.6 (10.26)	43.6–81.3	61.2 (11.06)	44.3–83.3
BMI	23.4 (2.35)	18.8–27.3	23.5 (2.18)	18.8–27.3	23.3 (2.56)	19.3–27.1

### Motivated behavior

For the evaluation of carry-over effects, the statistical test for comparing outcome distribution of the two groups at baseline in the second period was not significant (*p* = 0.96), indicating no carry over effect. This was to be expected, as the trial had planned a long-enough wash-out of 28 days between the two periods of the cross-over trial.

During the blinded data review an indication of period effect was noted and properly registered as follow up action according to the Statistical Analysis Plan of the trial protocol. As a mitigation strategy, if the test for period effect post unblinding was significant, then the statistical model for the primary outcome had to be recomputed only on the data from the first period of the trial (where period effect is taken out)—as recommended by clinical practice (35–37).

The test indicated a significant period effect (*p* < 0.001), mostly driven by learning and familiarization effects (both groups at baseline in the second group showed a higher performance rate than in the first period), as often seen in repetitive cognitive testing (38). Therefore, the clinical trial has been analyzed based on data from period one, with period 2 serving as supportive data.

**Figure 4:**
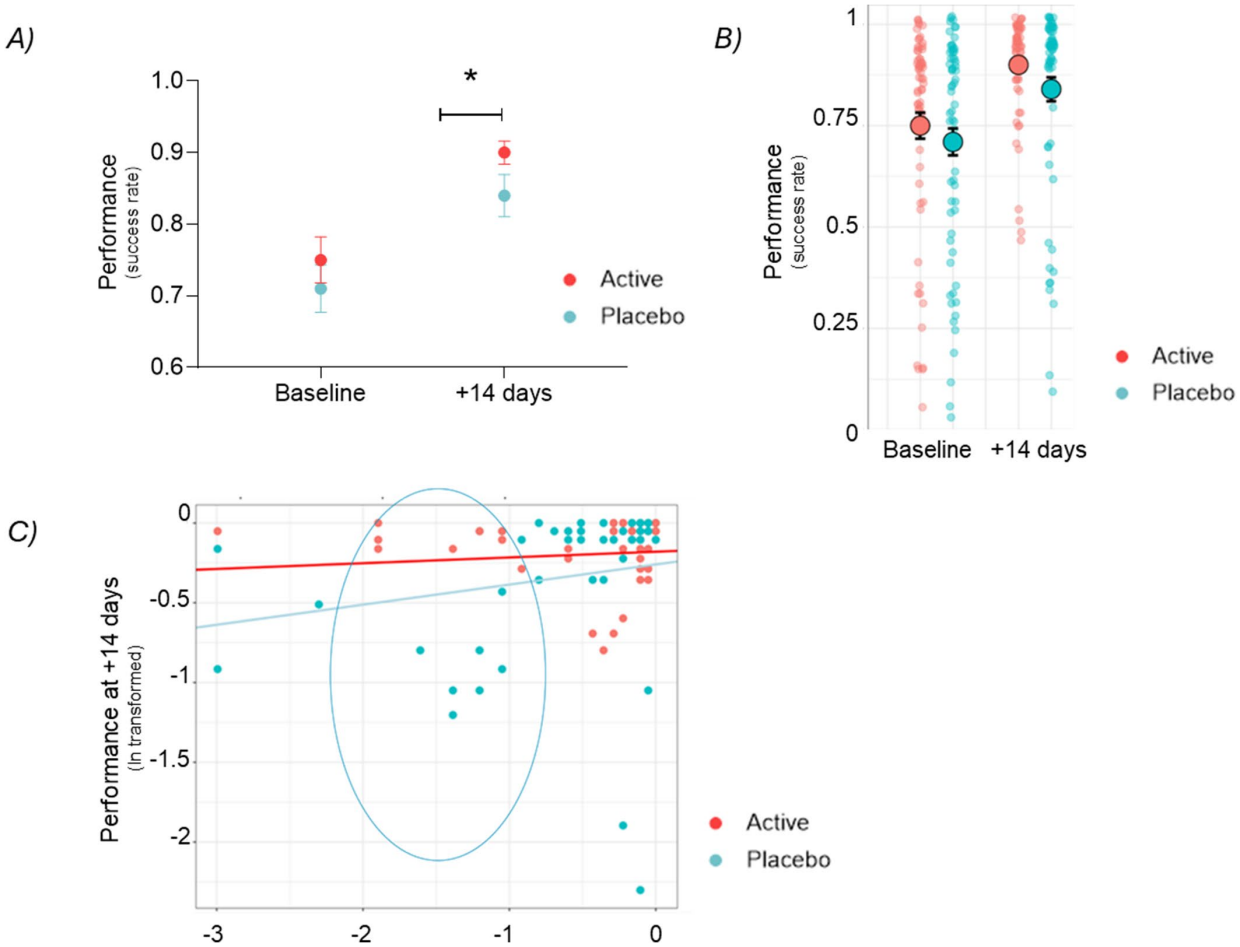
**(A)** Motivational performance as measured by success rate in incentivized trials at baseline and after 14 days (Visit 2) for the placebo and active (taurine, vitamin B6, B9, and B12) groups. Mean ± SEM, *p* = 0.039. **(B)** Motivational performance as measured by success rate in incentivized trials at baseline and after 14 days (Visit 2) for the placebo and active (taurine, vitamin B6, B9, and B12) groups. Shown as dot plot each dot is a participant. **(C)** Scatterplot of the MIFT success rate in incentivized trials after 14 days (*y*-axis) and at baseline (*x*-axis) for the placebo and the active group. Log transformation is used to normalize the data (no-normal distribution).

We observed a significant improvement in the performance in the active group compared to the placebo in the incentivized trials at +14 days after baseline (= monetary reward signal shown) (see Figure 4A).

Despite the observed improvement due to the learning and familiarization effect, the active group still showed a further +12% improvement on average [95% CI (1, 23%)] compared to the placebo group (*p* = 0.039). By plotting individual points (see Figure 4B) it is possible to observe a notable reduction of the lower tail in the active group, while the overall population shifts towards the upper limit of the performance range.

This conclusion is substantiated by an exploratory analysis of MIFT performance in relation to baseline measurements from the incentivized trials (see Figure 4C). The data suggest that the effect size of the treatment compared to the placebo increases as the baseline measurements decrease, thereby distancing itself from the ceiling effect observed in the data, which is limited by the number of trials conducted in this task. The model derived from the available data predicts that subjects receiving the active treatment, who had low baseline scores, will achieve on average higher scores at Visit 2 after 14 days of treatment compared to those in the placebo group. For instance, with a baseline MIFT score of 22%, the average predicted MIFT performance after 14 days is 87% [95% CI (68, 110%)] in the active group, while the placebo group average is predicted to reach only 70% [95% CI (57, 89%)], further emphasizing the effect of the active treatment. The highlighted section within the ellipse in Figure 4C illustrates the range of the above example. Because the two prediction intervals overlap, there is no statistical significant difference (*p* = 0.276).

The measurement at 4 weeks was not significant due to the ceiling effect of the learning and familiarization effect where the median of both groups was at 100% performance (Active group mean = 94%, SD = 0.09, median = 1.00; Placebo group mean = 94%, SD = 0.08, median = 1.00).

After the washout period of 4 weeks, although the performance decreased from the last visit in period 1, the new baseline performance has a median of 95% success (mean 90%)—in this case the placebo did not show a further improvement, while the active group still showed a significant improvement over the placebo after 28 days of product intake [estimate effect 8%; 95% CI (1–15%); *p* = 0.034] (see Supplementary Figure 4A) and again with a stronger effect on people with lower performance at baseline – highlighting the ceiling effect that may downsize the effect of the treatment (see Supplementary Figures 4B,C).

To further confirm the robustness of the results we also fitted the initially planned statistical model in which we considered data from periods and included the period as a fixed covariate. The improvement observed at 14 days of intake over placebo in the first period remained significant in incentivized trials [estimate effect 11%; 95% CI (2–20%); *p* = 0.016] as well as in no-incentivized trials [estimate effect 21%; 95% CI (3–40%); *p* = 0.026] and the effect sizes of all the findings are consistent with the analysis performed separating data by period (Supplementary Tables 4, 5). Nonetheless, as expected due to the important differences in baseline performance between the two periods, the combined periods’ effect is not significant.

**Figure 5:**
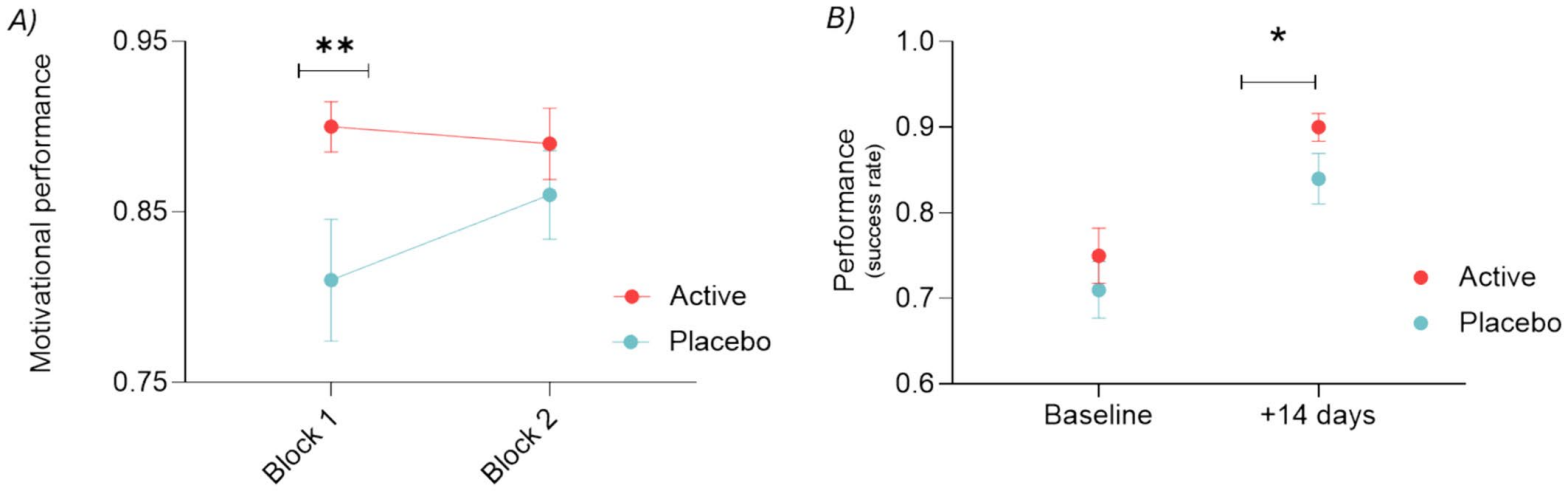
**(A)** Motivational performance as measured by success rate in block 1 and block 2, which are divided by 3 min break, for the placebo and active (taurine, vitamin B6, B9, and B12) groups during Visit 2 of period 1 (+14 days). **(B)** Motivational performance as measured by success rate normalized to the total number of trials at baseline and after 14 days (Visit 2) for the placebo and active (taurine, vitamin B6, B9, and B12) groups in the non-incentivized trials.

Interestingly, we have seen in previous research from Zalachoras et al. (17), that elevated levels of accumbal GSH can enhance motivated stamina over time in both humans and rats. This finding is comparable to the glutamine/glutamate ratio observed by Strasser et al. (16). As in these studies, the MIFT task was conducted in two sessions, with a three-minute break between them. Notably, when participants were in the active condition receiving the blend they were able to sustain consistent performance throughout the entire task (block 1 and block 2), exemplifying the concept of stamina which reflects the ability to maintain stable performance from beginning to end [estimate effect block 1 23%; 95% CI (10–37%); *p* = 0.001] (see Figure 5A). The same observation was noted during visit 3 of period 2, where the active group not only demonstrated superior performance, but also showed an enhanced ability to sustain that performance across blocks [see Supplementary Figure 5 estimate effect 11.7%; 95% CI (1–22%); *p* = 0.031].

According to the literature on motivation in effort-incentivized tasks, participants’ performance is expected to be higher during incentive trials compared to non-incentivized trials, reflecting the cost–benefit relationship (i.e., effort-reward) involved in taking action and performing. Indeed, performance in non-incentivized trials is significantly lower than in incentivized trials and all incentive levels different than the null (i.e., 10, 20, and 50 pesos respectively) are statistically significant different with respect to the zero-incentive level at 5% level (Table 2).

**Table 2:** Estimated incentive effect.

Contrast	Estimate	SE	df	*t*-ratio	*p*-value
0 pesos–10 pesos	−0.1463095	0.0197216	668.4279	−7.4187294	0.0000000
0 pesos–20 pesos	−0.1502634	0.0198198	669.4961	−7.5814631	0.0000000
0 pesos–50 pesos	−0.1584197	0.0201937	673.2465	−7.8449927	0.0000000
10 pesos–20 pesos	−0.0039539	0.0185180	652.0485	−0.2135187	0.8309891
10 pesos–50 pesos	−0.0121102	0.0185593	652.7236	−0.6525145	0.5142991
20 pesos–50 pesos	−0.0081563	0.0185423	652.4461	−0.4398752	0.6601732

However, the active group demonstrated superior performance compared to the placebo group after 14 days even in the non-incentivized trials of the task [estimate effect 22%; 95% CI (1–43%); *p* = 0.041] (see Figure 5B). This finding supports the concept of stamina, as it indicates that the active group maintained higher levels of performance even in the absence of incentives. We also examined the effect of sex, which was found to be non-significant (*p* = 0.693).

### Sustained attention

In the study, we included the PVT to measure sustained attention/concentration/focus as a secondary outcome.

**Figure 6:**
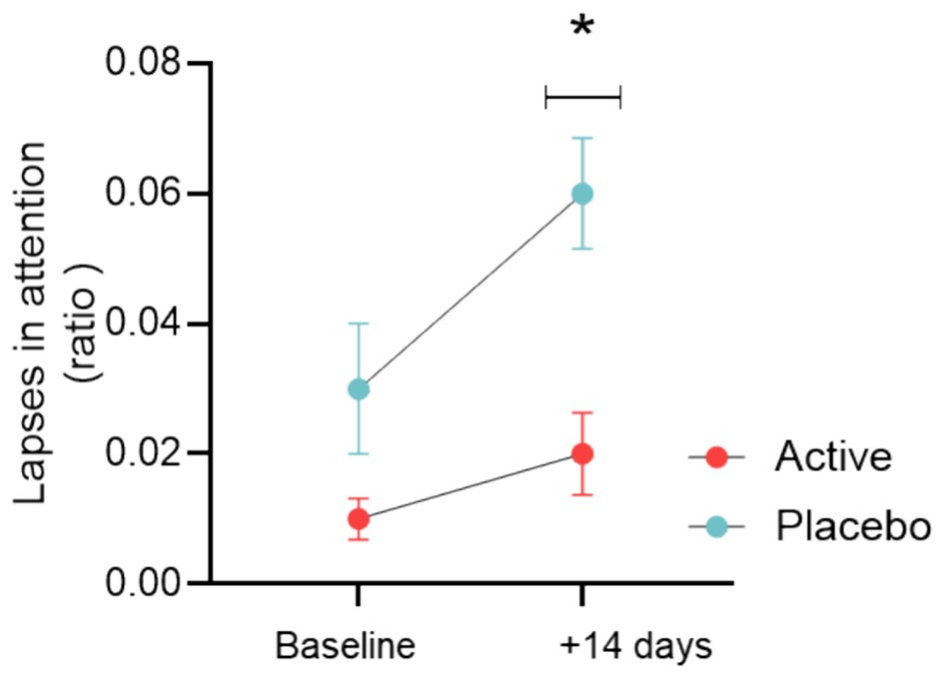
Attentional performance as measured by number of lapses normalized to the total number of trials at V1 (baseline) and after 14 days (Visit 2) for the placebo and active (taurine, vitamin B6, B9, and B12) groups. Median ± error of the median, *p* = 0.032.

As noted for the primary outcome MIFT, we observed a period effect (*p* = 0.027) linked to the familiarization effect. In this case, the performance declined until a plateau as people switched on spending resources to achieve a reward versus a no-rewarded paradigm (MIFT vs. PVT)—as also seen for incentive vs. no-incentivized trials in the MIFT. Nonetheless, results from the linear mixed model showed that the treatment effect was statistically significant for the PVT number of lapses at V2 (*p* = 0.032) (see Figure 6). This suggest that after 14 days of product intake in the first period (e.g., when all participants were still “naïve” to the procedures) the active group exhibited on average less omission rates than the placebo group, as measured by the number of lapses in the PVT (lack of attention).

### Perceived workload

**Figure 7:**
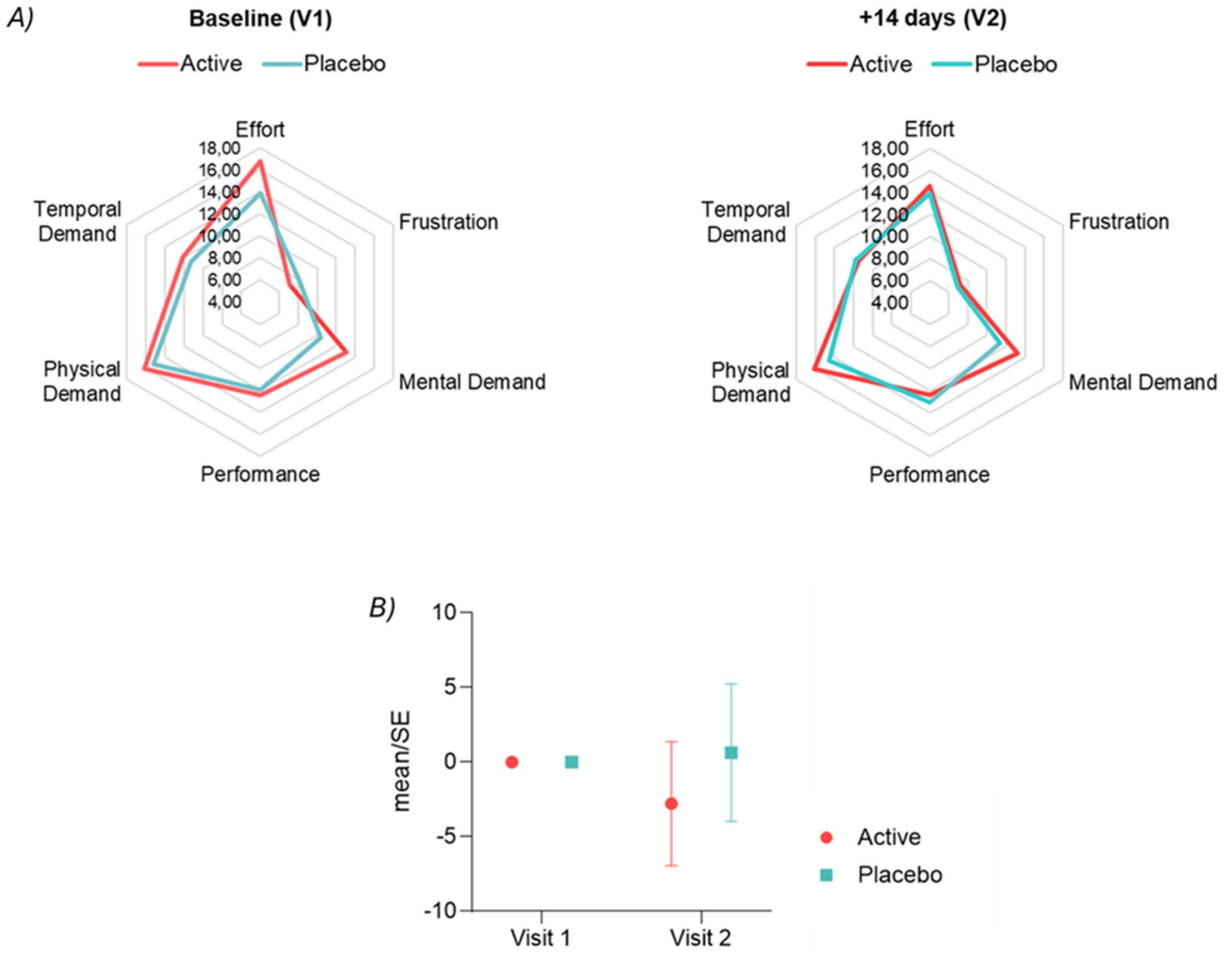
**(A)** Radar plot of the 6 domains of the NASA (after MIFT) questionnaire at Visit 1 (baseline) and at Visit 2 (after 14 days of product intake). **(B)** Delta NASA-TLX means standard error after MIFT from Visit 1 to Visit 2.

Radar plots for the average scores of the six dimensions of the NASA questionnaire after MIFT at visit 1 and visit 2 are displayed in Figure 7A.

To test the treatment effect on perceived workload, a composite score was obtained for the questionnaire NASA-TLX after MIFT by the sum of the scores on each of the six sub-domains. Mixed models performed on both composite scores revealed that the estimated treatment effect was not statistically significant. This suggests that there are no significant differences between the placebo and active conditions in the overall subjective workload ratings for the MIF task (see Figure 7A), nor across the entire duration of the study (see Supplementary Table 6). It is also plausible that the effect size is too small to be detected in our sample size due to the variability we observed in the responses.

Although not statistically significant, the analysis of perceived workload changes from baseline (Visit 1) to 14 days (Visit 2) is shown in Figure 7B.

### Mood of fatigue and vigor

We also investigated the feelings of Fatigue-Inertia and Vigor-Activity by using the short form of the Profile of Mood States (POMS) questionnaire to investigate if the supplementation may increase the perception of energy or reduction of fatigue experienced in the previous days.

The results revealed no statistically significant treatment effects in either domain at Visit 2 and 3 (14 and 28 days of intake), with *p*-values of 0.269 for Fatigue-Inertia and 0.553 for Vigor-Activity. While these findings suggest that there were no significant differences between the active and placebo groups regarding perceived levels of vigor and fatigue, caution is warranted when interpreting especially the Fatigue dimension. At baseline, the Fatigue-Inertia mean score was 1.84 for the active and 2.11 for the placebo condition, and the median score was 1 for both groups, indicating that the enrolled subjects did not exhibit significant fatigue issues that could have been improved. However, these scores also reflect values that deviate considerably from established normative mean values in the adult population from western cultures being reported to be 7.3 for men and 8.7 for women (39). On the same note, vigor values were quite high at baseline but closer to the normative values of 19.8 for men and 18.9 for women (39) (see Table 3).

**Table 3:** Descriptive statistics of the fatigue-inertia and vigor-activity mood dimensions at baseline and during the following visits.

			Active			Placebo		
Visit	Dimension	Records	Mean	SD	Median	Mean	SD	Median
V1 (baseline)	Fatigue-Inertia	44	1.84	2.72	1.0	2.11	3.17	1.0
V1 (baseline)	Vigor-Activity	44	14.32	4.62	15.0	13.91	4.93	14.0
V2 (+14 days)	Fatigue-Inertia	44	1.98	3.15	1.0	1.70	2.60	0.0
V2 (+14 days)	Vigor-Activity	44	14.36	3.96	15.0	13.84	5.19	14.0
V3 (+28 days)	Fatigue-Inertia	44	1.59	1.91	1.0	1.75	3.00	1.0
V3 (+28 days)	Vigor-Activity	44	14.59	4.18	15.5	14.05	4.71	15.0

### Blood levels of taurine and vitamins B6, B9, and B12

**Figure 8:**
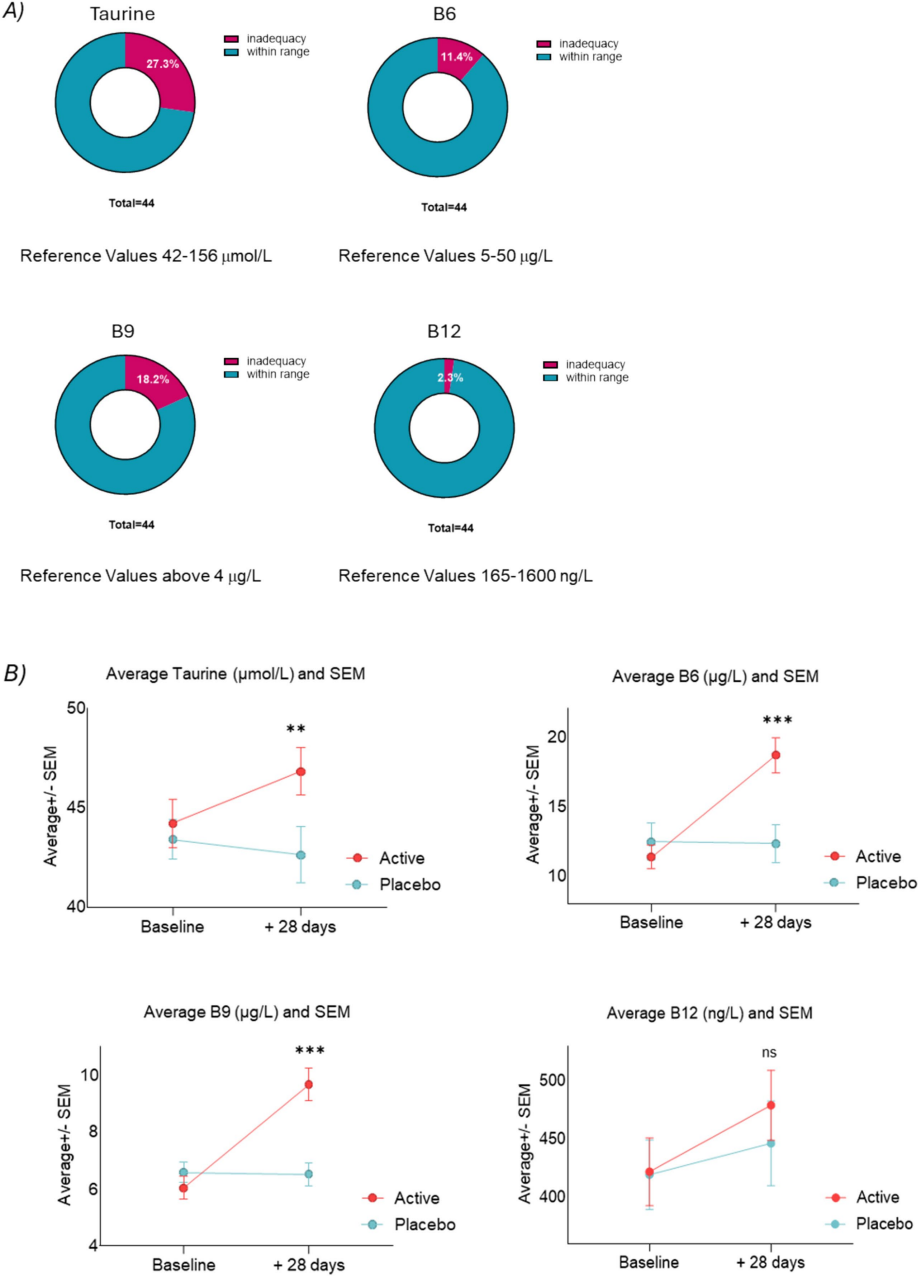
**(A)** Inadequacy of blood levels of taurine, B6, B9, and B12 in the subjects enrolled in the clinical study at baseline—laboratory reference values from Mayo Clinics and Metametrics Laboratory, Philippines. **(B)** Line plots of average taurine (µmol/L), vitamin B6 (µg/L), vitamin B9 (µg/L), and vitamin B12 (ng/L) measurements at V1 and V3.

We conducted an analysis of blood levels of taurine, vitamin B6, B9, and B12, revealing notable inadequacies within the population at baseline, particularly for taurine and B9 (see Figure 8A). Literature indicates that the prevalence of inadequacies in B vitamins among Filipinos may be even greater (40). Importantly, our preclinical work has established the significance of maintaining adequate levels of B9 to enable taurine to effectively increase GSH (see Supplementary Figure 1), which is considered a key driver of our mode of action. Results from mixed linear models showed that our supplementation led to a significant increase in blood levels of taurine (*p* = 0.006), B6 (*p* = 0.001), and B9 (*p* = <0.0001). However, no significant difference was observed in B12 (*p* = 0.384) levels between the placebo and our treatment groups, seemingly due to an increase in B12 levels also within the placebo group (see Figure 8B).

## Discussion

In healthy populations, mental performance exhibits a range of nuances that can be influenced by a balanced diet and a healthy lifestyle. While many individuals may appear to be functioning optimally, underlying factors such as stress, sleep quality, and nutritional inadequacies can affect their mental performance levels. To provide effective support to enhance motivated performance, it is essential to identify and understand the specific mechanisms that can be targeted to tailor dietary interventions. We began with the hypothesis that enhancing brain antioxidant mechanisms, particularly through the production of GSH, could improve motivated performance. In this study, we developed a targeted blend of taurine, vitamins B6, B9, and B12, aiming to achieve a synergistic effect that would support cognitive performance. Our *in vitro* experiments suggested that inadequacies in essential nutrients, such as vitamin B9, hinder the ability of taurine to effectively increase GSH levels in neural cells (see Supplementary Figure 1). Consequently, this limitation reduces taurine’s efficacy in protecting mitochondria from oxidative stress and maintaining adequate energy production when needed such as during prolonged tasks (see Supplementary Figure 2). This finding underscores the importance of a comprehensive approach to nutrient supplementation. Interestingly, our clinical study revealed that in the enrolled healthy adult population, the two nutrients most frequently found to be inadequate were indeed taurine and vitamin B9 (see Figure 8A) out of the four measured. Our supplementation effectively increased blood levels of taurine, vitamin B9, vitamin B6 and vitamin B12 among participants (see Figure 8B). Nonetheless, an increase in vitamin B12 was observed in both the placebo and active group resulting in non-statistically significant differences between the two groups for this nutrient.

A key finding of our study is that the active group showed superior performance in incentivized trials (see Figure 4), as well as non-incentivized trials (see Figure 5B), highlighting the effectiveness of the supplementation in enhancing motivation and cognitive stamina. The findings reveal a notable improvement in the active group compared to the placebo, with an average performance increase of +12% in incentivized trials, which compares well to existing interventional data. Indeed, in the study conducted by Li et al. (41) focused on improving motivation through real-time fMRI-based self-regulation of the nucleus accumbens, 19 participants under rtfMRI showed faster reaction time in the standard MIDT (no effort coupled). Similarly, Zhang et al. (42) observed an improvement in the reaction time in the MIDT reward trial following 5 weeks green tea supplementation in 46 healthy subjects (24 placebo and 22 active) (42). Moreover, the exploration of baseline effect suggests that the treatment effect size increases as baseline measurements are lower, indicating that the supplementation may be particularly beneficial for individuals with lower initial performance levels (see Figure 4).

One must consider that the crossover study design employed in our trial, featuring repetitive cognitive measurements at relatively short intervals within a healthy population, presents certain caveats. One notable concern is the potential confounding factor of learning and familiarization, which can result in pronounced period effects and ceiling effects in performance measurements. Given that we were unaware of the specific speed of learning within this population, we proactively addressed this risk by incorporating an analysis of these effects into the statistical analysis plan of the trial. This approach ensures that we can adequately account for any learning-related influences on cognitive performance, thereby enhancing the robustness and validity of our findings. Relatedly, despite the ceiling effect of the task due to the learning observed at four weeks, the active group continued to show significant improvements over the placebo at 28 days in the second period (after washout), especially among participants with lower baseline performance (Supplementary Figure 4). We might hypothesize that this sustained improvement may also contribute to long-term motivational resilience. Future studies should explore this effect over extended periods with reduced repetition to better understand its potential impact. Finally, one important consideration in crossover designs for nutritional interventions is the effectiveness of the washout period in minimizing carryover effects during subsequent administration periods. In this study, we examined the distribution of B vitamins and taurine across all visits, and only for vitamin B9 there was a slight suspicion of a carry-over effect, as V4 measurements were marginally higher in the placebo group than in the active group (*p* = 0.056). For all other blood markers, no evidence of carryover effects was identified, supporting the adequacy of the four-week washout period.

Overall, the study’s findings align with the hypothesis that supplementation with nutrients able to enhance GSH levels can improve stamina, as observed in both observational and preclinical studies. Indeed, another key finding is that, when receiving the active blend, participants demonstrated more consistent performance throughout the task, maintaining steady levels without fluctuations. Furthermore, the higher ability of participants to perform effectively even in non-incentivized trials may further suggest their greater stamina, indicating that the supplementation not only enhances performance under motivational incentives, but may also support sustained effort (see Figure 5). Interestingly, when subjects did not fully allocate their resources to the incentivized task those in the active group exhibited better sustained attention compared to the placebo group. This is evidenced by a reduced number of attentional lapses in the PVT, as illustrated in Figure 6. These results align well with previous studies and observations (43, 44). Importantly, higher intake of B vitamins is significantly associated with the ability to sustain attention as measured in the Digit Symbol Substitution Test (DSST) as well as in the Stroop Test (45). Consistently, a meta-analysis suggests that B vitamins supplementation is associated with slowing cognitive decline, especially in populations who received early intervention and intervention of long duration (46). A review of available evidence suggests that the effects of B vitamins supplementation can be beneficial for different cognitive domains, including attention, and that this effect is primarily evident in older adults and clinical populations (47). A study that investigated the associations between dietary history of past taurine intake and cognitive function in the elderly found that the average taurine index of the elderly with dementia (104.7 points) was significantly lower than average taurine index of the healthy elderly (123.7 points, *p* < 0.01) (48). There were positive correlations between total taurine index and total score of cognitive function in all the elderly subjects (*p* < 0.05) (48). To the best of our knowledge, no studies have explored the potential interconnection between taurine and vitamin B9. Evidently, our preclinical study indicates that this relationship may be crucial in driving certain mechanisms of action that influence brain health and performance (GSH synthesis and antioxidant defense).

With respect to mood measures (i.e., vigor and fatigue), the enrolled participants exhibited no signs of fatigue at baseline and demonstrated high vigor levels, comparable to those observed in Filipino athletes (athlete = 13.16, non-athlete = 12) (49), with no further improvement achievable. Notably, the values observed in this study differ significantly from the normative ones (see POMS results section). Additionally, while a comprehensive validation of the POMS has not yet been conducted in the Philippines, as it has for other languages and cultures (39, 50), at least one study utilizing POMS with a Filipino population reported results that were more consistent with the expected values for this age group (49). We have observed a significant prevalence of inadequacies in taurine, vitamin B6 and B9, with the overall average of the population residing at the lower end of the adequate range. It is well recognized that low levels of these micronutrients can lead to signs of fatigue (51, 52). For instance, in a human observational study, the level of taurine in the nucleus accumbens has been found to be inversely correlated to trait anxiety and state physical fatigue (52). Importantly, trait anxiety seems to negatively impact attentional control (53, 54), processing efficiency (55) and incentive processing when rewards require effortful exertion (6). These findings align with existing evidence on the relation between brain levels of GSH and psychogenic stress, with taurine being associated with both factors. For a comprehensive review, please refer to Zalachoras et al. (18). Collectively, this evidence strengthens the potential connection between our supplementation and its ability to enhance motivational performance and attention, while we could expect also to influence perceptions of fatigue and vigor. Therefore, further studies should explore the benefits of our supplementation in a population reporting signs of fatigue and/or lower vigor.

Furthermore, taurine has recently garnered attention as possible biomarker of aging, a complex and debated area within the scientific community. Indeed taurine has been observed to decline with age, and supplementation in different species seemed to improve health span (56, 57). However, more recent studies challenge the notion that taurine levels consistently decrease after the age of 26, as longitudinal data from large cohorts do not support this finding (58). It is essential to recognize that while there is no agreement on specific nutrients that drive aging, this does not negate the potential role of key nutrients and their supplementation in supporting a healthy longevity journey, as suggested by systematic reviews and meta-analysis on the benefit of taurine supplementation for cardiometabolic and oxidative stress parameters (59–62). Nutritional needs may vary significantly across different organs, and it is crucial to consider the intricate interactions between various nutrients as well as the different needs during the aging journey. For instance, the combination of taurine with sufficient vitamin B9 (folate) may enhance the production of antioxidants such as GSH in the brain better than single nutrient supplementation.

Understanding the mechanisms that drive the phenotypes observed in the healthy population is crucial for elucidating the diverse ranges of behaviors associated with these traits. By gaining insights into the underlying processes, we can more effectively modulate the interactions between various nutrients and optimize supplementation strategies. The knowledge acquired from the present study may be used to not only enhance the efficacy of nutritional interventions, but also to allow for more personalized approaches to promote overall health and well-being. Future research should explore whether the beneficial effects observed so far become even more pronounced in clinically vulnerable populations—such as older adults, individuals with obesity, individuals presenting depressive symptoms or patients with metabolic or neurological conditions—who may exhibit greater oxidative stress and thus respond more strongly to targeted nutritional interventions. A key direction will be determining how overall brain metabolite profiles can be modulated through diet and specific supplementation strategies, not only with the current ingredients but also in combination with broader dietary patterns. To strengthen mechanistic understanding, advanced brain-imaging approaches—particularly MR spectroscopy quantification of glutathione—will be crucial to confirm whether peripheral changes translate into measurable improvements in central antioxidant capacity and brain health.

## Conclusion

In our study, we designed and tested a nutritional supplementation of taurine, vitamin B6, B9, and B12 demonstrating a positive impact on supporting motivation and attention, enhancing mental energy and facilitating the effort required to achieve goals after 14 days of daily intake. This approach may serve as a viable strategy for improving performance in the adult population. We recommend conducting further research to investigate the potential long-term benefits of this supplementation in healthy adult populations.

## Data Availability

The data that supports the findings of this study can be made available to qualified scientists upon reasonable request. Requests to access the datasets should be directed to LT, laura.trovo@rd.nestle.com.
