# Supplementary material for: A nutritional blend of taurine, vitamins B6, B9, and B12 improves motivated behaviors in healthy adults—a double-blinded randomized clinical trial
Source: Front Nutr. 2026 Mar 11;13:1711478. doi: 10.3389/fnut.2026.1711478 (PMC13015822; doi:10.3389/fnut.2026.1711478)
Supplement: Supplementary file 1 [file Table_1.docx]

**Supplementary material – in vitro study**

***Rat primary cortical astrocytes***

Rat primary cortical astrocyte cells (N7745100, Life Technologies Europe BV, Bleiswijk, The Netherlands) of E19 Sprague Dawley rats were used for all experiments. After thawing, cells were centrifuged once at 300 g for 5 min in culture medium and cultured in uncoated T75 flasks (734–1067, VWR) in D-MEM without B9 (SIGMA/D2429) with the addition of glucose 3.5gr/L, L-glutamine 0.584 gr/L, NaHCO3 3.7 gr/L and with 15% fetal bovine serum (FBS; 16000–036, Life Technologies Europe BV) and 1 % penicillin–streptomycin (P4333, Sigma Aldrich, St. Louis, MO, USA).

For all tests, primary cortical astrocytes were treated for 48 h at 37 °C with different concentrations of ingredients individually or in combination, see Supplementary table 1.

Controls contained the corresponding volume of vehicle without the bioactive(s).

***In vitro GSH measurements***

Intracellular GSH was measured in rat primary astrocytes in culture by using the GSH-Glo™ Assay, which is a luminescent-based assay for the detection and quantification of reduced and/or total glutathione levels in cells. The assay is based on the conversion of a luciferin derivative into luciferin in the presence of GSH. The reaction is catalyzed by a glutathione S-transferase enzyme supplied in the kit. The luciferin formed is detected in a coupled reaction using Ultra-Glo™ Recombinant Luciferase that generates a glow type luminescence that is proportional to the amount of glutathione present in cells. A standard curve was used for each biological replicate and at least 5 technical replicates were done for each condition and for each biological replicate (3 biological replicates). 10% of lysate of each technical and biological replicate was used to measure protein amount by bicinchoninic acid assay (BCA) and used to normalize the GSH intracellular content to total protein amount. Buthionine sulfoximine (BSO), a specific inhibitor of γ-glutamylcysteineligase was added at 15 μM at the time of the start of the treatment to confirm the specificity of the readout.

Measurements were performed 48 hours after treatment. Results were compared to the control condition (vehicle) for each biological replicate and for each condition.

***Seahorse assay for mitochondrial fitness***

Primary astrocytes were cultured on Seahorse XF-96 (Seahorse BioSciences- Bucher Biotec AG) plates coated with poly-D-lysine: cat N° A3890401_ Gibco at 0.1mg/ml diluted at 50ug/ml with phosphate-buffered saline, at a density of 15000 cells per well. The cells were cultured in the medium described before and treated 48 hours before the oxidative stress application and metabolic flux as shown in Table 1.

We performed a “mitochondrial stress test” as per standard design (1), three baseline measurements of oxygen consumption rate (OCR) were sampled prior to sequential injection of mitochondrial inhibitors. Three metabolic determinations were sampled following the addition of each mitochondrial inhibitor prior to injection of the subsequent inhibitors. The mitochondrial inhibitors used were oligomycin (1.5 μm), FCCP (carbonyl cyanide 4-(trifluoromethoxy)- phenylhydrazone) (3 μm), and antimycin (5 μm) and rotenone (0.5 μm). OCR was automatically calculated and recorded by the Seahorse software. After the assays, cells were stained with DAPI to perform a nuclear counting for normalization using ImageXpress Micro Confocal High-content Imaging system from Molecular Devices (OCR values /cell). Oxidative stress (induced by tert-Butyl Hydroperoxide (tBHP) to astrocytes and mitochondrial function were investigated by performing a dose response of tBHP for 1 hour (7.8; 15.6; 31.2; 62.5; 125; 250 micromolar). 125μM tBHP was chosen to impact mitochondrial function and test the protective effects of the mentioned compounds. The spare respiratory capacity was calculated by the Seahorse assay as (Maximal Respiration) / (Basal Respiration) × 100, and coupling efficiency was calculated as ATP Production Rate) / (Basal Respiration Rate) × 100. In the treatment assays the values reported were normalized to vehicles.

***Statistical analysis***

The tissue culture in vitro data were performed 4 times (biological replicates) with at least 3 technical replicates for each condition. Data were analyzed by repeated measure one-way ANOVA with the Geisser-Greenhouse correction followed by Tukey’s multiple comparison test using GraphPad Prism 10.2.3.

**Reference**

1. Gu X, Ma Y, Liu Y, Wan Q. Measurement of mitochondrial respiration in adherent cells by Seahorse XF96 Cell Mito Stress Test. STAR protocols. 2021;2(1):100245.

**Supplementary method – clinical study**

**Participants’ Exclusion Criteria:**

- Any significant ongoing or past medical (including celiac disease, obstructive sleep apnea, restless leg syndrome) and/or psychiatric condition, which in the opinion of the site physician/investigator may compromise participant wellbeing/safety, impede participant compliance with study procedures, or ability to complete the study.
- Any clinically significant abnormality detected by the site physician/investigator during physical examination conducted at screening.
- Participants with reported intermediate visual acuity less than 20/25 and without correction.
- Known history of allergy to the ingredients in the investigational products.
- Participants that have taken part in another interventional clinical trial within the last 3 months.
- Current regular smoker (regularity defined ≥2 cigarettes per week).
- Pregnant, lactating, or intending to conceive during the clinical trial.
- Use of chronic over the counter or prescription medicines that may affect cognitive and physical functioning within 30 days or 5 half-lives prior to enrolment e.g., antidepressants, anxiolytics, antihistamines, narcotic analgesics.
- Alcohol consumption above daily recommended alcohol intake for men (2 drinks = 24 g/day) and women (1 drink = 12 g/day) (assessed by self-report; local guidelines)
- Caffeine consumption above recommended caffeine daily consumption, defined as > 400 mg/day (approximately 4 cups of coffee), and evaluated using the Caffeine Consumption Questionnaire.
- Consumption of energy drinks containing Taurine and/or Vitamin Bs within 21 days prior to enrolment
- Use of vitamin Bs and/or Whey protein and/or amino acid supplements within 30 days of enrolment.
- Presence of sleep disorders (evaluated using the global sleep assessment questionnaire (GSAQ))
- Performing shift work or trans-meridian travel within 10 days of enrolment

**FIGURES**

**Supplementary Figure 1:** Glutathione increase in astrocytes after 48 hours treatment with different doses of taurine and relative B9 ratios (1:2500; 1:700 and taurine alone), normalized to their vehicle (Mean, SEM) and BSO inhibition to confirm the signal is specifically related to the synthesis of GSH.


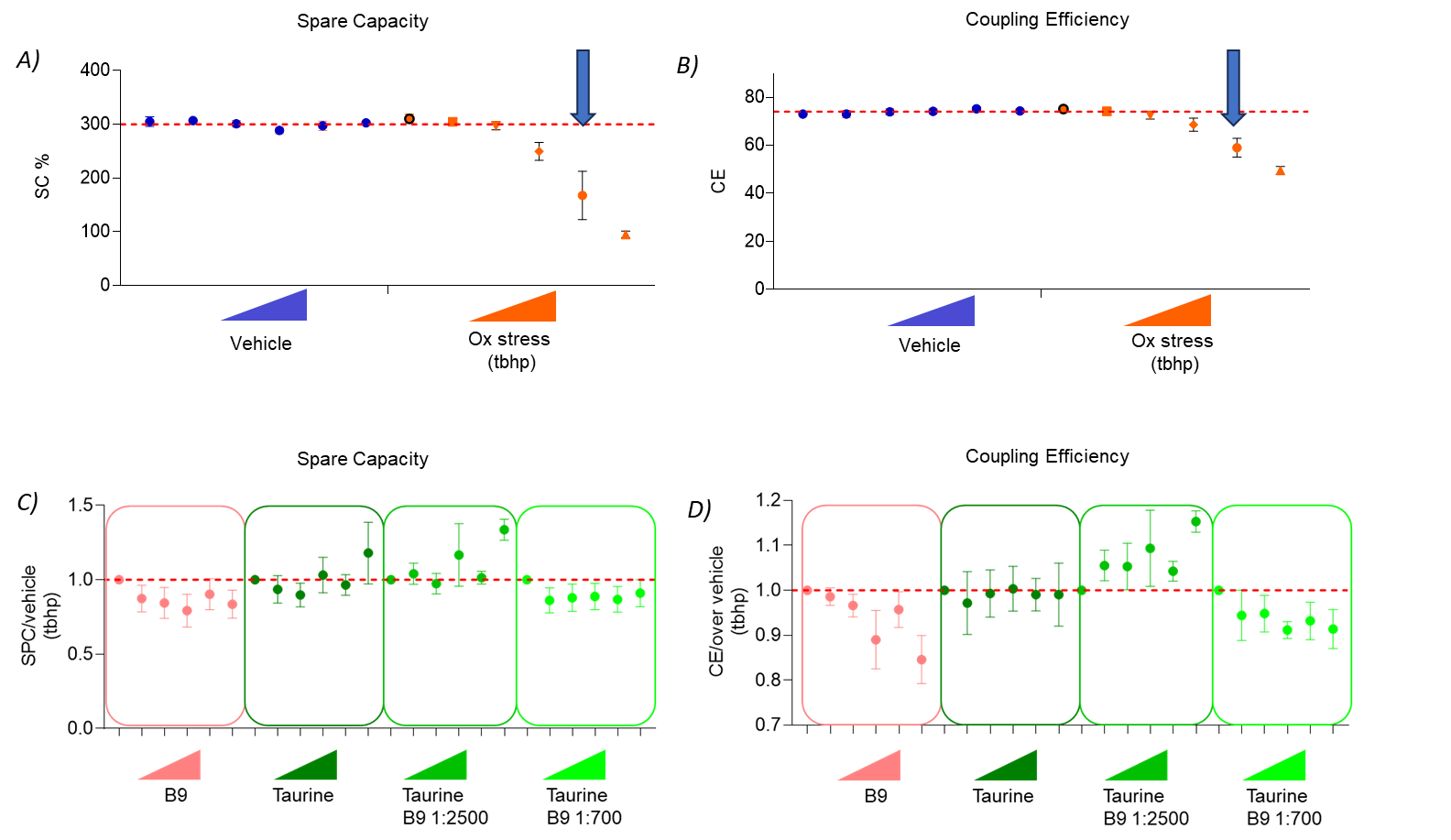


**Supplementary Figure 2**. Panel A and B: A dose-response experiment was conducted using tert-Butyl hydroperoxide (tBHP) for one hour prior to the Seahorse measurements. This approach was utilized to determine the effective dose that influences mitochondrial fitness assessed through mitochondrial spare capacity and coupling efficiency – The arrows denote the dosage chosen for evaluating blend protection in following experiments. The doses tested were 7.8, 15.6, 31.2, 62.5, 125, and 250 micromolar. Panel C and D: Protection of mitochondrial fitness assessed via spare capacity and coupling efficiency in astrocytes after 48 hours treatment with different doses of taurine and relative B9 ratios (1:2500; 1:700 and taurine alone), normalized to their vehicle (Mean, SEM)

**
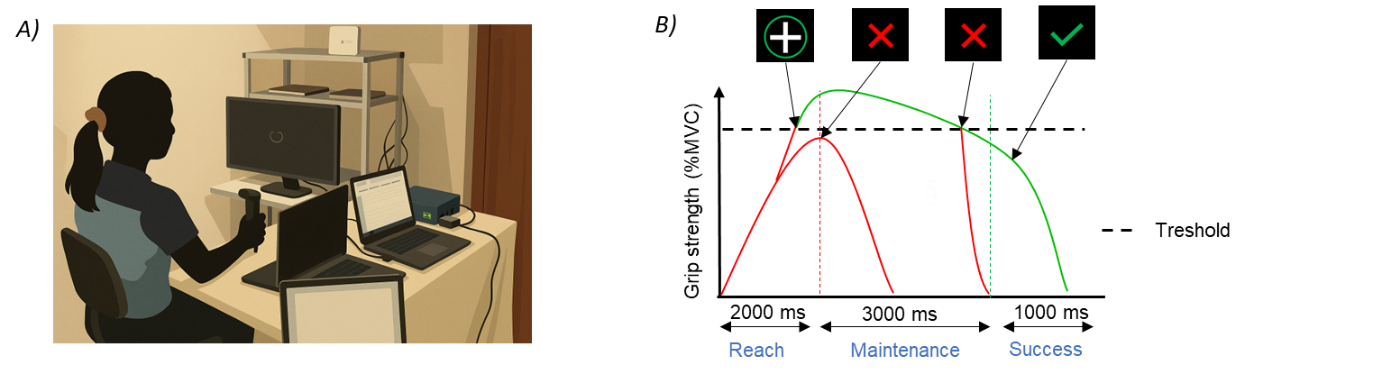
Supplementary Figure 3.** Panel A: Graphical representation of the lab set-up for the MIF task. Panel B: possible outcomes and relative trajectories for each trial: a green circle appeared around the fixation cross if the threshold was reached within 2 seconds; a red cross appeared if participants failed to reach the threshold in the initial 2 sec or if the force level fell below the maintenance threshold during the 3 sec maintenance period; a green tick appeared If the force was maintained for the required 3 sec.

**
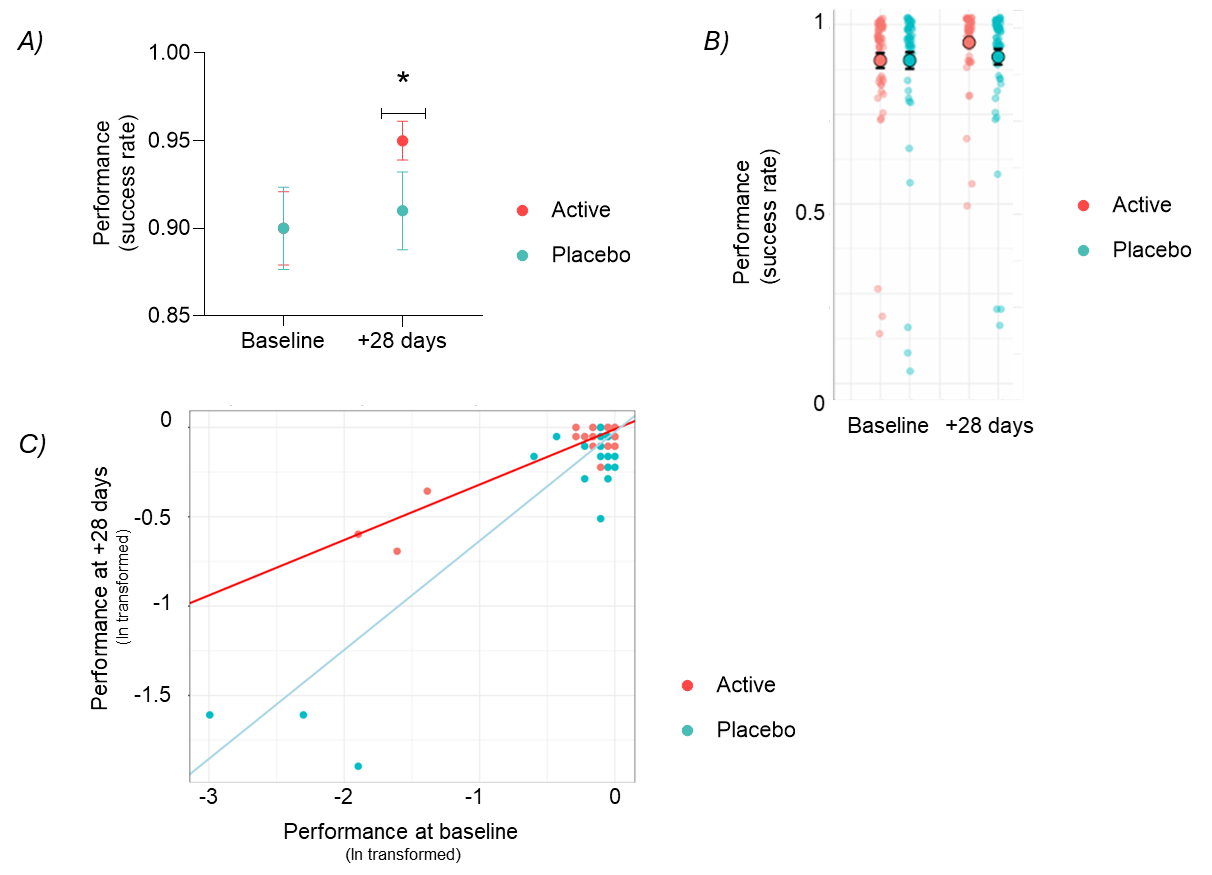
**

**Supplementary Figure 4.** Panel A: performance as measured by success rate normalized to the total number of trials at baseline and after 28 days in the second period -supportive data- for the placebo and active (Taurine, Vit B6 B9 and B12) groups. Mean +/- SEM, p= 0.034. Panel B: Motivational performance as measured by success rate in incentivized trials at baseline, after 14 days (Visit 2), at 28 days (Visit 3) in the second period for the placebo and active (Taurine, Vit B6 B9 and B12) groups. Shown as dot plot each dot is a participant. Panel C: Scatterplot of the MIFT success rate after 28 days (y-axis) and at baseline (x-axis) for the placebo and the active group in the second period. Log transformation is used to normalize the data (no-normal distribution).

**
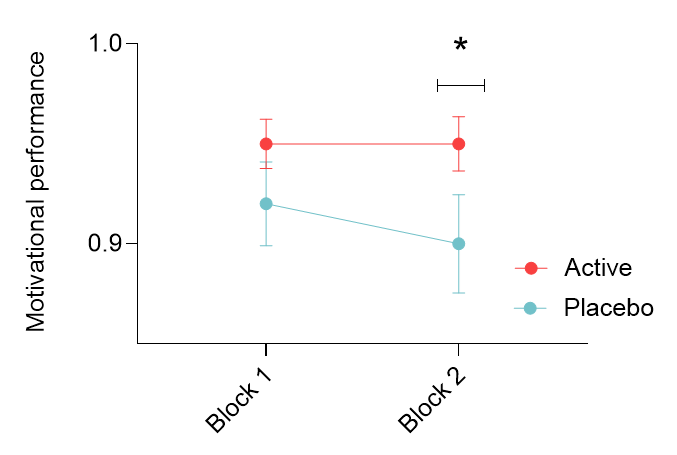
**

**Supplementary Figure 5.** Motivational performance as measured by success rate in block 1 and block 2, which are divided by 3 minutes break, for the placebo and active (Taurine, Vit B6 B9 and B12) groups during Visit 3 of period 2.

**TABLES**

**Supplementary Table 1.** The amounts and ratios of taurine and vitamin B9 used in the comparative analysis are shown, where one ratio contains 3.5 times more vitamin B9 (1:700) than the other ratio (1:2500).

|  | Ratio 1:2500 | Ratio 1:700 |
| --- | --- | --- |
| Taurine (𝞵M) | Vit B9 (𝞵M) | Vit B9 (𝞵M) |
| 2000 | 0.8 | 2.9 |
| 667 | 0.27 | 0.95 |
| 222 | 0.09 | 0.32 |
| 74 | 0.03 | 0.11 |
| 25 | 0.01 | 0.04 |
| 8 | 0.003 | 0.012 |
| 3 | 0.001 | 0.004 |

**Supplementary Table 2:** Listing of concomitant medications (N=4).

| **Treatment** | **Category** | **Route** | **N** |
| --- | --- | --- | --- |
| Vitamin C (Immuno Pro) | Supplement | Oral | 1 |
| Ethinyl Estradiol, Levonorgestrel | Other | Oral | 1 |
| Ebastine, Betamethasone | Other | Oral | 1 |
| Ethinyl Estradiol, Levonorgestrel, Ferrous Fumarate | Other | Oral | 1 |

**Supplementary Table 3:** Description of reported adverse events (N=25).

| **Preferred Term** | **Relationship** | **#AE** | **# Subjects** |
| --- | --- | --- | --- |
| Somnolence | Probable | 5 | 5 |
| Pain in extremity | Related | 43 | 20 |
| Nasopharyngitis | Unlikely | 1 | 1 |
| Back pain | Unrelated | 2 | 1 |
| Somnolence | Unrelated | 2 | 2 |
| Headache | Unrelated | 1 | 1 |

43 adverse events (declared by 20 subjects, approximately 50% of the total recruited subjects in the study) are categorized as “Pain in extremity” for preferred term (but the term declared by the subjects was “momentary pain on hand grip”) and it was related to the study procedure (i.e. MIFT hand grip dinamometer). 5 subjects declared somnolence and it is probable related to the Investigational product or study procedures.

**Supplementary Table 4:** Estimated treatment effect for incentivized trials of the MIFT.

| **Contrast** | **Estimate** | **SE** | **confidence interval** | **p-value** |
| --- | --- | --- | --- | --- |
| log(Active/Placebo) at period 1, V2 | 0.11095 | 0.04538 | [0.02, 0.2] | 0.016 |
| log(Active/Placebo) at period 1, V3 | -0.03025 | 0.04538 | [-0.12, 0.06] | 0.506 |
| log(Active/Placebo) at period 2, V2 | -0.01261 | 0.04533 | [-0.1, 0.08] | 0.781 |
| log(Active/Placebo) at period 2, V3 | 0.08044 | 0.04533 | [-0.01, 0.17] | 0.079 |

| **Contrast** | **Estimate** | **SE** | **confidence interval** | **p-value** |
| --- | --- | --- | --- | --- |
| Active - Placebo at period 1, V2 | 0.21495 | 0.09463 | [0.03, 0.4] | 0.026 |
| Active - Placebo at period 1, V3 | -0.02142 | 0.09463 | [-0.21, 0.17] | 0.821 |
| Active - Placebo at period 2, V2 | -0.06131 | 0.09436 | [-0.25, 0.13] | 0.518 |
| Active - Placebo at period 2, V3 | -0.02722 | 0.09436 | [-0.21, 0.16] | 0.774 |

**Supplementary Table 5:** Estimated treatment effect for no-incentivized trials of the MIFT.

**Supplementary Table 6:** Perceived Workload after MIFT at each visit for the placebo and active group.

| **Visit** | **Treatment** | **Records** | **Min** | **Q1** | **Median** | **Q3** | **Max** | **Mean** | **Sd** |
| --- | --- | --- | --- | --- | --- | --- | --- | --- | --- |
| Visit 1 | Active | 22 | 28 | 67.25 | 80.5 | 88.75 | 110 | 77.73 | 19.66 |
| Visit 1 | Placebo | 22 | 42 | 60.25 | 70.0 | 80.00 | 103 | 70.86 | 16.52 |
| Visit 2 | Active | 22 | 39 | 63.00 | 76.5 | 89.00 | 104 | 74.95 | 19.49 |
| Visit 2 | Placebo | 22 | 20 | 62.50 | 73.5 | 79.00 | 120 | 71.50 | 21.58 |
| Visit 3 | Active | 22 | 31 | 61.50 | 80.5 | 90.00 | 100 | 75.68 | 18.85 |
| Visit 3 | Placebo | 22 | 25 | 65.00 | 73.5 | 85.75 | 120 | 72.73 | 22.62 |
| Visit 4 | Active | 22 | 22 | 65.50 | 71.5 | 89.75 | 120 | 74.41 | 23.91 |
| Visit 4 | Placebo | 22 | 47 | 66.50 | 75.0 | 91.00 | 114 | 78.32 | 16.71 |
| Visit 5 | Active | 22 | 20 | 69.00 | 75.5 | 86.50 | 100 | 72.73 | 20.16 |
| Visit 5 | Placebo | 22 | 43 | 63.50 | 76.0 | 85.00 | 106 | 75.23 | 16.62 |
| Visit 6 | Active | 22 | 22 | 63.00 | 75.5 | 86.50 | 120 | 72.55 | 23.52 |
| Visit 6 | Placebo | 22 | 38 | 60.75 | 73.5 | 87.50 | 119 | 73.59 | 20.45 |

**Supplementary Table 7:** Descriptive statistics of the POMS dimensions and total score at baseline and during the following visits.

|  | | | | | Active |  |  |  | Placebo |  |  |
| --- | --- | --- | --- | --- | --- | --- | --- | --- | --- | --- | --- |
| Visit | Dimension | | records | mean | sd | median |  | mean | sd | median |  |
| V1 (baseline) | Anger - Hostility | | 44 | 1.43 | 2.75 | 0.0 |  | 2.20 | 2.80 | 1.0 |  |
| V1 (baseline) | Confusion - Bewilderment | | 44 | 2.11 | 2.74 | 1.0 |  | 2.45 | 3.39 | 1.0 |  |
| V1 (baseline) | Depression - Dejection | | 44 | 0.68 | 1.39 | 0.0 |  | 1.23 | 2.17 | 0.0 |  |
| V1 (baseline) | Friendliness | | 44 | 14.23 | 4.04 | 15.0 |  | 13.30 | 4.23 | 13.5 |  |
| V1 (baseline) | Tension - Anxiety | | 44 | 1.91 | 2.84 | 1.0 |  | 2.68 | 3.11 | 2.0 |  |
| V1 (baseline) | Total Mood Disturbance | | 44 | -6.34 | 12.37 | -10.0 |  | -3.23 | 13.90 | -6.0 |  |
| V2 (+14 days) | Anger - Hostility | | 44 | 1.34 | 2.18 | 0.0 |  | 1.91 | 2.38 | 1.0 |  |
| V2 (+14 days) | Confusion - Bewilderment | | 44 | 1.75 | 2.31 | 1.0 |  | 1.80 | 2.24 | 1.0 |  |
| V2 (+14 days) | Depression - Dejection | 44 | | 0.59 | 1.00 | 0.0 |  | 0.75 | 1.50 | 0.0 |  |
| V2 (+14 days) | Friendliness | 44 | | 14.07 | 3.42 | 14.5 |  | 13.20 | 4.31 | 13.5 |  |
| V2 (+14 days) | Tension - Anxiety | 44 | | 1.64 | 2.14 | 1.0 |  | 1.98 | 2.96 | 1.0 |  |
| V2 (+14 days) | Total Mood Disturbance | 44 | | -7.07 | 10.45 | -9.0 |  | -5.70 | 12.30 | -7.0 |  |
| V3 (+28 days) | Anger - Hostility | 44 | | 1.41 | 2.07 | 0.0 |  | 1.41 | 1.99 | 0.0 |  |
| V3 (+28 days) | Confusion -Bewilderment | 44 | | 1.86 | 2.62 | 1.0 |  | 1.68 | 2.49 | 1.0 |  |
| V3 (+28 days) | Depression - Dejection | 44 | | 0.61 | 1.26 | 0.0 |  | 0.77 | 1.46 | 0.0 |  |
| V3 (+28 days) | Friendliness | 44 | | 14.16 | 3.40 | 14.5 |  | 13.55 | 3.84 | 15.0 |  |
| V3 (+28 days) | Tension - Anxiety | 44 | | 1.57 | 1.90 | 1.0 |  | 2.00 | 2.44 | 1.0 |  |
| V3 (+28 days) | Total Mood Disturbance | 44 | | -7.55 | 10.46 | -10.0 |  | -6.43 | 12.14 | -9.5 |  |
